# Serum Lipid Profiles, Lipid Ratios and Chronic Kidney Disease in a Chinese Population

**DOI:** 10.3390/ijerph110807622

**Published:** 2014-07-29

**Authors:** Liying Zhang, Zhiyong Yuan, Wu Chen, Shanying Chen, Xinyu Liu, Yan Liang, Xiaofei Shao, Hequn Zou

**Affiliations:** 1 Department of Nephrology, The Third Affiliated Hospital of Southern Medical University, Guangzhou 510630, China; E-Mails: zhangliying926@126.com (L.Z.); dqydqy386@sina.com (S.C.); pepsi84@163.com (X.L.); lsky2008@yeah.net (Y.L.); shaoxfei@126.com (X.S.); 2 Department of Nephrology, The First Affiliated Hospital of Inner Mongolia Medical University, Hohhot 010000, China; 3 Wanzai Hospital of Xiangzhou district, Zhuhai 519000, China; E-Mail: wzyyyzy@sina.com; 4 Qianjin health service center of Tianhe district, Guangzhou 510000, China; E-Mail: dragonchw@sina.com; 5 Department of Nephrology, Zhangzhou Affiliated Hospital of Fujian Medical University, Zhangzhou 363000, China

**Keywords:** serum lipids, lipid ratios, chronic kidney disease

## Abstract

*Aim:* To examine the association of serum lipids, lipid ratios with Chronic Kidney Disease (CKD) in a Chinese population. *Methods:* Data were drawn from a cross-sectional survey in China. CKD was defined as estimated glomerular filtration rate (eGFR) < 60 mL/min/1.73m^2^ or albuminuria-to-creatinine ratio (ACR) > 30 mg/g. Multivariable logistic regressions and multivariate regression models were used. Serum lipids and lipid ratios included total cholesterol (TC), triglyceride (TG), low-density lipoprotein cholesterol (LDL-C), high-density lipoprotein cholesterol (HDL-C), TG/HDL-C ratio, TC/HDL-C ratio and LDL-C/HDL-C ratio. *Results:* In men, only logarithm-transformed (log) TG was associated with CKD. The odds ratio (every SD increment) was 1.39 (95% CI 1.03–1.87, *P* = 0.03). In women, none of the serum lipids and lipid ratios was associated with CKD. Using multivariate regression models, it was shown that log TG and log TG/HDL-C were negatively correlated with eGFR (*P* < 0.05) in men and LDL-C and log LDL-C/HDL-C ratio were correlated with ACR in men. In female subjects, serum TC, log TG, log TG/HDL-C and log TC/HDL-C were negatively correlated with eGFR (*P* < 0.05). All of serum lipid profiles and lipid related ratio were not correlated with ACR in women. *Conclusion:* Serum TG is the only suitable predictor for CKD in men. However, in women, none of serum lipids and lipid ratio can be used as a predictor for CKD. Log TG and log TG/HDL-C are negatively correlated with eGFR in both genders.

## 1. Introduction

It is well recognized that serum lipids are linked to atherosclerotic diseases [1]. Chronic kidney disease (CKD) shares some common risk factors, such as hypertension and diabetes, with cardiovascular diseases. Serum lipids might be independent risk factors for CKD. The results of previous studies are inconsistent [2,3,4,5,6]. A study by Kurella *et al.* [2] indicated that each component of metabolic syndrome including hypertriglyceridemia and low high density lipoprotein cholesterol (HDL-C) is an independent risk factor of developing CKD. Previous studies based on Korean populations indicated that triglyceride (TG)/HDL-C ratio is independently associated with CKD [3,4]. However, in two previous studies based on Chinese populations, neither hypertriglyceridemia nor low HDL-C is associated with CKD [5,6].

As we know, there are limited data on the association of serum lipids with CKD in Chinese populations. Therefore, the association of serum lipids and CKD needs to be further explored in Chinese populations. The aim of the current study is to examine the association of serum lipid profiles, lipid ratios with CKD in a Chinese population.

## 2. Experimental Section

### 2.1. Study Population

This population-based, cross-sectional survey was conducted in Wanzai Town, Zhuhai City located in Southern China. The cross-sectional survey has been described in our previous paper [7]. In the current study, we briefly described the methods of the study again. We randomly selected three communities in Wanzai Town to perform the survey. This survey was conducted from June 2012 to October 2012. We invited all residents aged 18 years or older to participate in this survey. In total, 2142 study subjects (the mean age was 50 ± 13 years) participated in the survey and 308 subjects were excluded due to missing data. In the current study, 1834 subjects were included [7].

### 2.2. Ethics Statement

This study was approved by the ethics committee of the Third Affiliated Hospital of Southern Medical University. Written informed consents were given by all subjects.

### 2.3. Serum Lipid Profiles and Lipid-Related Ratios

Serum total cholesterol (TC), serum TG, and serum HDL-C were determined using colorimetric methods with the Roche assay (Roche cobas6000). Serum low density cholesterol (LDL-C) was indirectly calculated.

Lipid ratios are also used to predict cardiovascular risks and the ratios might be the stronger predictors of heart disease [8,9]. In the current study, we also used lipid ratios to predict the risk of CKD. TG/HDL-C was calculated as TG divided by HDL-C. The other two lipid ratios were TC/HDL-C ratio and LDL-C/HDL-C ratio.

According to the Chinese guideline, the optimal serum lipid profile is serum total TC level < 5.18 mmol/L, TG level < 1.7 mmol/L, LDL-C level < 3.37 mmol/L, and HDL-C level ≥ 1.04 mmol/L [10]. Dyslipidemia is defined by the presence of at least one of the following: serum total TC level ≥ 5.18 mmol/L, TG level ≥ 1.7 mmol/L, LDL-C level ≥ 3.37 mmol/L, and HDL-C- cholesterol level < 1.04 mmol/L, and/or having received treatment for dyslipidemia during the previous 2 weeks [10]. 

### 2.4. Determination of CKD

Both albuminuria and decreased glomerular filtration rate were used as markers of kidney damage. First morning void urine samples were collected. Women who were actively menstruating and subjects having urinary tract infection symptoms were excluded from the urine test. Urinary albumin was measured by an immune nephelometric method with the Orion assay. Serum creatinine and urinary creatinine were determined using colorimetric methods with the Roche assay [7].

Estimated glomerular filtration rate (eGFR) was calculated as 175× (Scr)−1.234 × (Age)−0.179 × (if female, × 0.79). This is a modified MDRD equation which has been used as an alternative method to assess eGFR in the Chinese population [11]. Urinary albumin to creatinine ratio (ACR, mg/g) was calculated as urinary albumin divided by urinary creatinine. A decreased eGFR was defined as eGFR less than 60 mL/min/1.73m^2^. CKD was defined as a decreased eGFR and/or ACR ≥ 30 mg/g [12].

### 2.5. Data Collection

All members of trained medical staff, including physicians, general practitioners, medical students and nurses, performed physical examinations and data collection according to standardized procedures. Data on demographic status, personal and family history, education attainment, and lifestyle were obtained through questionnaires [7]. 

A full physical examination included anthropometric measurements and blood pressure measurement. According to the World Health Organization recommended procedures, height, weight, waist circumference and hip circumference were measured [13]. Waist circumference was accurate to 0.1 cm and weight was accurate to 0.1 kg. Systolic blood pressure (SBP) and diastolic blood pressure (DBP) were determined three times on the right arm in a seated position using a calibrated mercury sphygmomanometer. The average of the three consecutively reading was recorded [7].

Blood specimens were collected after an overnight fast for at least 10 hours and stored at 2 °C–8 °C until analysis. All samples were analyzed in the central laboratory of Third Affiliated Hospital of Southern Medical University [7].

Serum lipids, serum creatinine, serum fasting glucose, serum insulin and sensitivity C-reactive protein (CRP) was determined.

### 2.6. Data Analysis

All statistical analyses were conducted using Stata (version 11). Statistical significance was set at *P* value < 0.05. We used mean ± standard deviation to describe continuous variables with a normal distribution and medians and interquartile ranges for skewed distributed variables. Frequencies and percentages were used to indicate categorical variables.

Clinical characteristics of the study population were listed. We also compared the characteristics of male and female subjects. Student’s T test or rank-sum test were used for continuous variables and the chi-squared test for categorical variables.

In order to examine the associations of serum lipids, lipid ratios with CKD, logistic regression models were used for estimating the odds ratios (OR) and 95% confidence interval (CI). In the current study, the associations of serum lipids, and lipid ratios with CKD were explored in male and female subjects, respectively. The models were adjusted for socio-demographic status (age and educational attainment), comorbidities (history of hypertension, history of diabetes, and history of coronary heart disease and history of stroke), lifestyle factors (current smoking, current alcohol use, physical inactivity), systolic blood pressure (SBP), diastolic blood pressure (DBP), serum fasting glucose and waist circumference. Only eight subjects (five men and three women) were taking statins, so taking statin was not added as a covariate.

We also used multivariate regression models to examine the correlations of serum lipids and lipid ratios with eGFR or ACR in each gender, respectively. In the regression models, eGFR or logarithmically transformed ACR was used as a dependent variable, respectively. Multivariate regression models were also adjusted for variables which were used in the logistic regression models. These variables included age, educational attainment, history of hypertension, history of diabetes, history of coronary heart disease, history of stroke, current smoking, current alcohol use, physical inactivity, SBP, DBP, serum fasting glucose and waist circumference.

All logistic regression analyses and multivariate regression models were conducted separately in male and female subjects. All variables with a skewed distribution were logarithmically transformed before being analyzed. These variables included serum TG, TG/HDL-C ratio, TC/HDL-C ratio, LDL-C/HDL-C ratio and ACR.

## 3. Results

### 3.1. Clinical Characteristics of Study Population (Table 1)

A total of 1834 subjects (the mean age was 53 ± 15 years) were included in the analysis. Among 1834 subjects, only 37.02% (679) subjects were men and 12.81% (235) subjects had CKD.

Men had a significantly larger waist circumference, a higher SBP and a higher DBP than women. Women had a higher level of eGFR than men, but there was no significant difference on prevalence of CKD between men and women. In the past three months, no subjects used contrast agents or antibiotics. The proportions of current smokers and current alcohol use were significantly higher in men.

Men had a higher TG/HDL-C ratio, a higher TC/HDL-C ratio and a higher LDL-C/HDL-C ratio than women, and all of these differences were significant (*P* < 0.05). Men also had higher serum TGs and lower HDL-Cs than women.

**Table 1:** Clinical characteristics of male and female subjects.

	Total	Male	Female	*P* value
	*n* = 1834	*n* = 679	*n* = 1155	
Clinical Characteristics				
Age (Years)	53 ± 15	53 ± 15	53 ± 14	0.29
Waist circumference(cm)	83 ± 10	86 ± 10	81 ± 10	<0.001
History of Hypertension (%)	372 (20.28)	166 (24.45)	206 (17.84)	0.001
History of Diabetes (%)	115 (6.27)	48 (7.07)	67 (5.80)	0.29
History of Coronary heart disease (%)	42 (2.29)	23 (3.39)	19 (1.65)	0.27
Current smoker (%)	223 (12.16)	214 (31.52)	9 (0.78)	<0.001
Current alcohol use (%)	106 (5.78)	94 (13.84)	12 (1.04)	<0.001
Education attainment High school or above (%)	761 (41.49)	344 (50.66)	417 (36.10)	0.92
Physical inactivity (%)	1013 (55.23)	374 (55.08)	639 (55.32)	<0.001
Systolic blood pressure (mmHg)	129 ± 20	130 ± 19	127 ± 21	0.003
Diastolic blood pressure (mmHg)	78 ± 11	80 ± 11	77 ± 11	<0.001
Serum lipids				
Cholesterol (mmol/L)	5.39 ± 1.03	5.34 ± 0.99	5.42 ± 1.05	0.09
LDL (mmol/L)	3.18 ± 0.91	3.14 ± 0.92	3.20 ± 0.90	0.19
HDL (mmol/L)	1.54 ± 0.33	1.44 ± 0.31	1.60 ± 0.32	<0.001
Triglyceride (mmol/L)	0.65 (0.32–1.98)	1.37 (0.97–2.17)	1.15 (0.83–1.61)	<0.001
LDL/HDL ratio	2.09 (1.67–2.59)	2.22 (1.81–2.76)	2.00 (1.60–2.48)	<0.001
TG/HDL ratio	0.83 (0.53–1.35)	1.03 (0.63–1.68)	0.73 (0.48–1.17)	<0.001
TC/HDL ratio	3.58 (3.01–4.15)	3.83 (3.23–4.38)	3.45 (2.92–3.99)	<0.001
Other labratory				
Serum creatitine (umol/L)	73 ± 17	87 ± 15	65 ± 11	<0.001
eGFR( mL/min/1.73m^2^)	99 ± 22	92 ± 20	104 ± 22	<0.001
ACR (mg/g)	8.49 (5.75–14.23)	9.54 (6.63–15.56)	6.81 (4.59–11.46)	<0.001
Fasting glucose (mmo/L)	5.01 ± 1.19	5.07 ± 1.26	4.98 ± 1.14	0.10
Serum C-reactive protein (mg/L)	0.99 (0.46–2.37)	1.01 (0.49–2.63)	0.98 (0.44–2.25)	0.17
CKD	235 (12.81)	83 (12.22)	152 (13.16)	0.56
Dyslipidemia	1474 (80.37)	556 (81.89)	918 (79.48)	0.21

TC: total cholesterol; TG: triglyceride; LDL-C: low-density lipoprotein cholesterol; HDL-C: high-density lipoprotein cholesterol; eGFR: Estimated glomerular filtration rate; ACR: Urinary albumin to creatinine ratio; CKD: chronic kidney disease.

### 3.2. Association of Serum Lipids, Lipid Ratios with CKD in Male and Female Subjects (Table 2)

In unadjusted models, TC, logarithm-transformed (log) TG, and log TG/HDL-C ratio were significantly associated with CKD in male subjects. After adjusted for age, educational attainment, comorbidities, lifestyle factors, blood pressure, fasting glucose and waist circumference, only log TG was associated with CKD. The odds ratio (every SD increment) was 1.39 (95% CI 1.03–1.87, *P* = 0.03).

Table 2 showed that in the unadjusted model, TC, log TG, log LDL-C/HDL-C ratio, log TG/HDL-C ratio and log LDL-C/HDL-C ratio were associated with the risk of incident CKD in female subjects. However, further adjusted for potential confounders, none of the serum lipids and lipid ratios was associated with CKD.

**Table 2:** Association of serum lipids and lipid ratios with Chronic Kidney Disease (CKD) in male and female subjects.

	Men				Women			
	Unadjusted		Adjusted *		Unadjusted		Adjusted *	
	OR (95% CI)	*P* value	OR (95% CI)	*P* value	OR (95% CI)	*P* value	OR (95% CI)	*P* value
**TC (one SD)**	1.29 (1.03–1.61)	0.03	1.21 (0.91–1.62)	0.19	1.27 (1.08–1.50)	0.004	0.97 (0.79–1.20)	0.79
**HDL (one SD)**	1.17 (0.94–1.44)	0.16	1.20 (0.91–1.59)	0.19	0.99 (0.84–1.18)	0.94	1.12 (0.93–1.36)	0.22
**LDL (one SD)**	0.97 (0.77–1.23)	0.80	0.89 (0.67–1.18)	0.40	1.18 (1.00–1.40)	0.054	0.92 (0.75–1.13)	0.44
**Log-triglyceride (one SD)**	1.53 (1.23–1.91)	<0.001	1.39 (1.03–1.87)	0.03	1.44 (1.22–1.69)	<0.001	1.03 (0.83–1.28)	0.82
**Log-TG/HDL (one SD)**	1.44 (1.14–1.82)	0.002	1.30 (0.95–1.77)	0.10	1.39 (1.17–1.64)	<0.001	0.98 (0.78–1.22)	0.83
**Log-TC/HDL (one SD)**	1.13 (0.90–1.43)	0.31	1.03 (0.76–1.40)	0.84	1.29 (1.08–1.54)	0.005	0.84 (0.67–1.06)	0.14
**Log-LDL/HDL (one SD)**	0.93 (0.74–1.17)	0.52	0.83 (0.64–1.09)	0.18	1.22 (1.02–1.46)	0.03	0.86 (0.69–1.07)	0.18

Adjusted for age, educational attainment, history of hypertension, history of diabetes, history of coronary heart disease, history of stroke, current smoking, current alcohol use, physical inactivity, systolic blood pressure, diastolic blood pressure, fasting glucose and waist circumference. TC: total cholesterol; TG: triglyceride; HDL-C: high density lipoprotein cholesterol; LDL-C: low density lipoprotein cholesterol. Log: logarithm-transformed; SD: standard deviation.

### 3.3. Correlation of eGFR or ACR with Serum Lipids and Lipid Ratios

Correlations of eGFR/ACR with serum lipids and lipid ratios in each gender were shown in Table 3. In the adjusted models, log TG and log TG/HDL-C were negatively correlated with eGFR in male subjects (*P* < 0.05). The coefficient and 95% CI for log TG were −4.89 (95% CI: −7.61, −2.17, *P* < 0.001). And the coefficient and 95% CI for log TG/HDL-C were −4.04 (95% CI: −6.53, −1.65, *P* < 0.001). LDL-C and log LDL-C/HDL-C ratio were correlated with ACR. The co-efficient and 95% CI were −0.08 (95% CI: −0.15–−0.01, *p* = 0.02) for LDL and −0.25 (95% CI: −0.43–−0.07, *p* = 0.007) for log LDL-C/HDL-C, respectively.

In female subjects, serum TC, log TG, log TG/HDL-C ratio and log TC/HDL-C ratio were negatively correlated with eGFR (*P* < 0.05). The respective coefficient and 95% CI were −1.45 (95% CI: −2.76–−0.15, *p* = 0.03) for log TC, −1.78 (95% CI: −3.14–−0.43, *p* = 0.01) for log TG, −1.57 (95% CI: −2.92–−0.22, *p* = 0.03) for log TG/HDL-C ratio, and −1.53 (95% CI: −2.87–−0.18, *p* = 0.03) for log TC/HDL-C, respectively. All of serum lipid profiles and lipid related ratio were not correlated with ACR.

**Figure 1:**
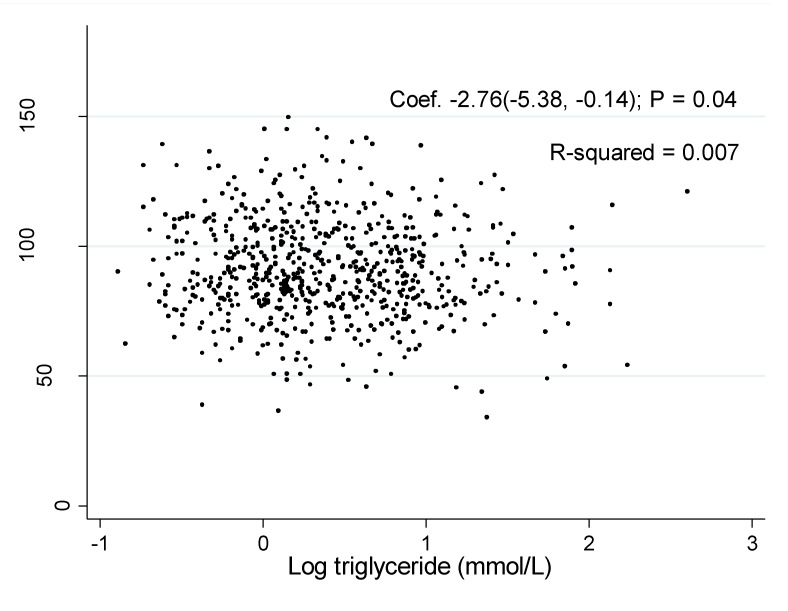
Association of estimated glomerular filtration rate with log triglyceride in men.

**Figure 2:**
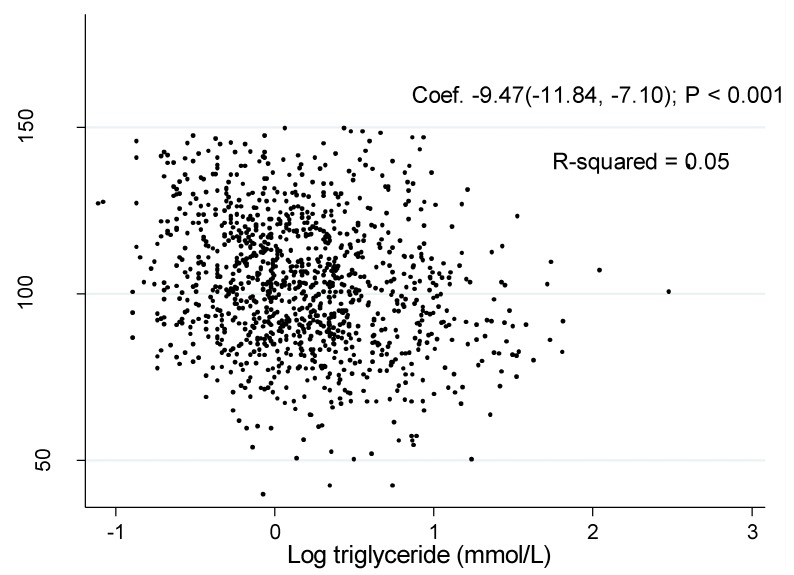
Association of estimated glomerular filtration rate with log triglyceride in women.

The associations of log TG with eGFR was shown in Figure 1 and Figure 2. The coefficient between log TG and eGFR in the unadjusted model were −2.76 (95% CI: −5.38, −0.14, *P* = 0.04) in men and −9.47 (95% CI: −11.84, −7.10, *P* < 0.001) in women, respectively. 

**Table 3:** Correlation of estimated glomerular filtration rate (eGFR) and urinary albumin to creatinine ratio (ACR) with lipid profiles in male and female subjects.

	Men				Women			
	eGFR		ACR		eGFR		ACR	
	Coefficient * (95% CI)	*P* value	Coefficient * (95% CI)	*P* value	Coefficient * (95% CI)	*P* value	Coefficient * (95% CI)	*P* value
**Total Cholesterol**	−0.82 (−2.30–0.67)	0.28	−0.03 (−0.10–0.03)	0.31	−1.45 (−2.76–−0.15)	0.03	−0.02 (−0.07–0.03)	0.43
**HDL**	−0.01 (−4.84– 4.84)	1.00	0.06 (−0.15–0.28)	0.55	0.17 (−1.05–1.39)	0.79	0.03 (−0.02–0.08)	0.18
**LDL**	0.30 (−1.28–1.88)	0.71	−0.08 (−0.15–−0.01	0.02	−0.74 (−2.03–0.55)	0.26	−0.03 (−0.08–0.02)	0.25
**Log-triglyceride**	−4.89 (−7.61–−2.17)	<0.001	0.003 (−0.12–0.12)	0.96	−1.78 (−3.14–−0.43)	0.01	0.005 (−0.05–0.06)	0.86
**Log-TG/HDL**	−4.04 (−6.53–−1.65)	0.001	−0.005 (−0.11–0.10)	0.94	−1.57 (−2.92–−0.22)	0.03	−0.006 (−0.06–0.05)	0.83
**Log-TC/HDL**	−5.65 (−12.64–1.35)	0.11	−0.21 (−0.51–0.10)	0.19	−1.53 (−2.87–−0.18)	0.03	−0.05 (−0.10–0.005)	0.08
**Log-LDL/HDL**	0.42 (−3.70–4.55)	0.84	−0.25 (−0.43–−0.07)	0.007	−0.64 (−1.93–0.65)	0.33	−0.03 (−0.08–0.02)	0.22

Adjusted for age, educational attainment, history of hypertension, history of diabetes, history of coronary heart disease, history of stroke, current smoking, current alcohol use, physical inactivity, systolic blood pressure, diastolic blood pressure, fasting glucose and waist circumference. TC: total cholesterol; TG: triglyceride; HDL-C: high density lipoprotein cholesterol; LDL-C: low density lipoprotein cholesterol. Log: logarithm-transformed; eGFR: Estimated glomerular filtration rate; ACR: urinary albumin to creatinine ratio

## 4. Discussion

The major finding of the current study was that the TG was associated with an increased risk of CKD in men. However, in women, none of the serum lipids and lipid ratios was associated with CKD. In both men and women, TG and TG/HDL-C ratio were negatively correlated with eGFR. In women, none of the serum lipids and lipid ratios was correlated with albuminuria. 

Several previous studies showed that both a high level of cholesterol and triglyceride might play an important role in the pathogenesis and progression of kidney disease [14,15,16,17,18,19,20,21,22]. In rat models, it was found that hypercholesterolemia can induce a pro-inflammatory response and result macrophages recruitment [17]. Both hyperlipidemia and macrophage influx appear to precede in the genesis of glomerulosclerosis [17,18,19,20,21,22]. In rats with type 2 diabetes, lipoxidation stress was also related to glomerulosclerosis and tubulointerstitial disease [18]. It seemed that food restriction can prevent hypertriglyceridemia induced glomerular injury and macrophage influx in obese Zucker rat [20]. Both hypercholesterolemia and hypertriglyceridemia are associated with podocyte injury which might be accompanied by tubulointerstitial injury [21,22]. All of these studies indicated that dyslipidemia can contribute to kidney damage. 

Dyslipidemia is an independent risk factor for progression of kidney disease in patients with diabetes [23]. In Samuelsson *et al.*’s study [24], 73 non-diabetic patients with CKD were followed for an average of 3.2 years. In the study, it is indicated that TC, LDL-C, and triglyceride-rich apoB-containing lipoproteins contributed to a more rapid decline in renal function. The results of the studies based on health persons were disparity [2,5,6]. After 9 years’ follow-up in the Atherosclerosis Risk in Communities study [2], it was shown that hypertriglycemia and low HDL-C were associated with the incident of CKD. However, the models were only adjusted for age, gender and race. Other potential confounders, such as blood pressure and serum glucose, were not added into the models [2]. One study based on 1456 elderly Chinese individuals indicated that low HDL-C and hypertriglyceridemia did not predict the risk of new onset of CKD [6]. In another cross-sectional study based on 4944 Chinese individuals, the association of low HDL-C and an elevated triglyceride level with CKD was abolished when adjusting for potential confounders [5]. In the current study, TG was associated with the incident CKD in men, but all of the serum lipids and lipid ratios were unlikely associated with CKD in women.

It has been recognized that small, density LDL-C phenotype is a risk factor of coronary heart disease [25]. The small, dense LDL-C phenotype is more commonly accompanied by the presence of hypertriglyceridemia, low HDL-C cholesterol levels, centrally obesity, and insulin resistance. The series of metabolic disorders might predict endothelial dysfunction that might lead to an increased susceptibility to thrombosis [26,27,28,29,30,31]. Reduction in fasting triglyceride levels will enlarge LDL-C particle size and reduce the risk of coronary heart disease [30]. However, a serum level of small density LDL-C particle is not a routine test in clinical practice. An alternation is using TG/LDL-C ratio which might be a surrogate for small, density LDL-C [32]. In one previous study, when using TG/HDL-C ratio to predict the existence of a small LDL-C particle size pattern, the sensitivity was 75.9% and the specificity 85.4% [33]. TG/HDL-C ratio can be used as a surrogate of insulin resistance and can be used to predict coronary heart disease independently [29,30,31,34]. Kim *et al.* used lipid ratios to predict CKD in Korean populations. The results indicated that TG/HDL-C ratio is the only lipid ratio associated with CKD in both men and women [3,4]. However, in the current study, we did not find significant association of lipid ratios with CKD in a Chinese population. 

Although, hyperlipidemia and hypercholesterolemia are independent risk factors for progression of kidney disease, no conclusive evidences demonstrate that isolated hyperlipidemia can lead to CKD in healthy individuals [22]. Hyperlipidemia might be accompanied by coronary heart disease, diabetes and hypertension. Recently, studies have demonstrated that statin therapy can halt the progression of kidney failure in patients with CKD, diabetes or coronary heart disease [35,36,37,38]. An elevated level of TG and a low level of HDL-C are also components of metabolic syndrome [39]. According to the results of one study based on a Chinese population, an increased number of metabolic syndrome components is associated with CKD [5]. These results support lipid-lowering therapy in patients with CKD, diabetes and coronary heart disease. In the current study, it was also shown that both TG and TG/HDL-C ratio were negatively correlated with eGFR in men and women. LDL-C and LDL-C/HDL-C were associated with albuminuria in men. 

Here, several limitations of the current study should be acknowledged. Firstly, this is only a cross-sectional study. Previous studies have shown that heavy proteinuria or end stage renal disease might be related with increased athrogenicity of LDL-C [40,41]. However, in the current study, only four subjects had macroalbuminuria (ACR > 300 mg/g) and no subjects had CKD 5 stage (eGFR < 15 mL/min). It was unlikely that dyslipidemia in this population was caused by CKD. Second, both albuminuria and eGFR were not measured repeatedly. Third, because we only included individuals who responded to an invitation, this might lead to a selection bias. Those individuals having more comorbidity might have a higher rate of participation willingness. In the current study, only 37.0% of subjects were men. 

## 5. Conclusions

Among serum lipids profiles and lipid ratios, only serum TG is a suitable predictor for CKD in men and the association is independent of other potential confounders. In women, none of the serum lipids and lipid ratios can be used as a predictor for CKD. Log TG and log TG/HDL-C are negatively correlated with eGFR in both genders. In men, LDL-C and log LDL-C/HDL-C ratio are correlated with ACR. In female subjects, no serum lipid or lipid ratio is correlated with ACR.
